# Clinical Utility of Thallium-201 Single Photon Emission Computed Tomography and Cerebrospinal Fluid Epstein-Barr Virus Detection Using Polymerase Chain Reaction in the Diagnosis of AIDS-Related Primary Central Nervous System Lymphoma

**DOI:** 10.7759/cureus.606

**Published:** 2016-05-10

**Authors:** Fadilah S Hussain, Namath S Hussain

**Affiliations:** 1 Radiology, Wayne State University School of Medicine; 2 Department of Neurosurgery, Penn State Hershey Medical Center

**Keywords:** thallium-201 spect, primary cns lymphoma, aids, hiv infection, brain biopsy, radiation therapy

## Abstract

Objective: To determine the diagnostic efficiency of thallium-201 single photon emission computed tomography (SPECT) and Epstein-Barr virus (EBV) polymerase chain reaction (PCR) in the differentiation of primary central nervous system lymphoma (PCNSL) from other central nervous system processes in patients with HIV/AIDS.

Design/Methods: Over 10 years, 68 thallium-201 SPECT scans were performed on neurologically symptomatic HIV+ patients with focal lesions on CT or MRI at the Johns Hopkins Hospital. Diagnoses were then established by either autopsy, biopsy, or clinical response to anti-toxoplasmosis therapy. Patients were categorized prior to a prospective clinical reading of the SPECT scans by nuclear medicine physicians.

Results: In our patient sample overall, the diagnostic efficiency of thallium-201 SPECT was 79%. The diagnostic accuracy of EBV PCR testing alone in a subset of 22 patients in our study that had CSF analyzed was 73%. However, when both positive EBV PCR and positive thallium-201 SPECT results were used together, the diagnostic accuracy improved to 100% based on a sample of 13 patients where EBV PCR and SPECT imaging results were concordant.

Conclusion: Thallium-201 SPECT has a relatively high positive predictive value with regards to the diagnosis of PCNSL, which suggests that patients with positive results could undergo empiric radiation treatment without resorting to brain biopsy. However, the predictive value can be increased by testing for CSF EBV using PCR. Alternatively, if CSF cannot be safely obtained because of mass effect, we believe that these data still suggest that empiric radiation treatment should be considered when discussing treatment options with patients with a positive thallium-201 SPECT.

## Introduction

The early and accurate diagnosis of mass lesions in patients with advanced human immunodeficiency virus (HIV) infection remains a challenging clinical problem. Cerebral toxoplasmosis, progressive multifocal leukoencephalopathy (PML), and primary CNS lymphoma (PCNSL) are the three most common causes of AIDS-related intracranial lesions in the United States. The CDC surveillance figures for 1997 reported toxoplasmosis as an AIDS-indicator condition in 4% of patients and in 1% of cases each of PCNSL and PML [1]. Specific MRI and CT imaging characteristics are relatively specific in identifying PML, including U-fiber subcortical involvement and the lack of enhancement and mass effect. However, despite some studies suggesting that certain CT or MRI characteristics, such as periventricular location [2], can reliably distinguish PCNSL from toxoplasmosis, generally, these imaging modalities are insufficient for diagnosis. Other diagnostic tests, like toxoplasma serologies, have also proven inadequate.

A test that is potentially useful is the detection of the Epstein-Barr virus (EBV) DNA in cerebrospinal fluid (CSF) by polymerase chain reaction (PCR), which appears to have a relatively high sensitivity and specificity for AIDS-related PCNSL [3]. Deluca, et al. have shown the test to be a sensitive and specific diagnostic tool. In their study, EBV DNA was found using PCR in 7/8 biopsy-proven cases of PCNSL and in 0/1 patients with other intracranial lesions, indicating a specificity of 100% and a sensitivity of 87.5% [4]. In another study of 20 patients with AIDS-related intracranial lesions undergoing stereotactic brain biopsy and CSF analysis, EBV PCR was shown to have a sensitivity of 100% [5]. Averaging across several studies, the overall sensitivity is 97% with a specificity of 98% and a positive predictive value of 90% [3-5].

The current standard of care dictates that patients presenting with contrast-enhancing lesions be treated empirically with anti-toxoplasmosis therapy. They are followed for two to four weeks and assessed for a clinical or radiologic response with CT or MRI. If there is no response, a stereotactic brain biopsy is performed. Only occasionally will the patient be treated empirically for lymphoma with radiation therapy. Early diagnosis of PCNSL is vital to maximize response to radiation treatment. The mean survival for untreated patients from the onset of symptoms has been reported to vary from 42 days [6] to 21 days [7] in other studies. Although brain biopsy has a reported diagnostic yield of 88%, the morbidity is 8% and the mortality is 3% in this patient population [7]. In addition, empiric treatment for toxoplasmosis may be associated with a drug reaction in up to 62% of patients and necessarily delays radiation treatment by at least two to four weeks [8]. Thus, more rapid and less invasive diagnostic techniques are needed to reduce this high complication rate and to facilitate earlier diagnosis. Thallium-201 is a potassium analog that accumulates in brain tumors but is not taken up by normal brain tissue or necrotic material, such as would be found in an abscess. Several studies have examined the use of thallium-201 SPECT in HIV patients with CNS mass lesions [9-11]. These studies have shown the test to have a high sensitivity and specificity but have been limited by small sample sizes. Herein, we determined the clinical utility of thallium-201 SPECT by examining a larger patient sample.

## Materials and methods

Brain thallium SPECT imaging was performed using a three-headed gamma camera and ultra-high resolution collimator, beginning approximately 15 minutes after intravenous injection of 3 mCi thallium-201.

Over 10 years, 68 thallium-201 SPECT scans were performed on neurologically symptomatic HIV+ patients with focal lesions on CT or MRI (49 males, 19 females) at the Johns Hopkins Hospital. Diagnoses were then established by either autopsy (4), biopsy (14), clinical response to anti-toxoplasmosis therapy (20), or clinical course (30). The gold standard for diagnosis of PCNSL is via pathologic examination of a biopsy specimen.

Patients were categorized prior to a prospective clinical reading of the SPECT scans by experienced nuclear medicine physicians. All cross-sectional images were reviewed and directly compared to CT or MRI when available. Images were interpreted as positive when activity in the area of interest was greater than the scalp or the contralateral brain region.

## Results

The alternative diagnoses included toxoplasmosis, progressive multifocal leukoencephalopathy, septic emboli, stroke, meningitis, brain abscess, and tuberculosis. Twenty out of 26 patients with PCNSL had focal areas of thallium uptake corresponding to CT/MRI lesions. Eight out of 42 patients with other diagnoses had false-positive scans. Thirty-four out of 42 patients without PCNSL showed no thallium uptake on scanning. Six out of 26 patients with PCNSL had false-negative scans.

The sensitivity of the thallium-201 SPECT for PCNSL was 77%, with a specificity of 81%. A positive scan had a predictive value of 71% while a negative scan had a predictive value of 85%. Overall, the diagnostic accuracy was 79%. The diagnostic accuracy of EBV PCR testing alone in a subset of 22 patients in our study that had CSF analyzed was 73%. However, when both EBV PCR and thallium-201 SPECT results were used together, the diagnostic accuracy improved to 100% based on a sample of 13 patients where the EBV PCR and SPECT imaging results were concordant.

## Discussion

In an attempt to more clearly delineate the clinical utility of thallium-201 SPECT in identifying AIDS patients with CNS mass lesions, we examined the diagnostic accuracy of the test in determining whether or not patients have PCNSL as opposed to other common etiologies for mass lesions in AIDS patients, such as cerebral toxoplasmosis. We found the diagnostic accuracy of the test to be somewhat lower than reports from previous studies [9-10]. These studies reported no false-negative scans. However, another recent study of nine patients with PCNSL did report three false-negative scans [12]. There is also a case report in the literature describing a study where a patient with PCNSL did not, in fact, have any increased radioisotope uptake on a thallium-201 SPECT scan [13]. False-negative scans have been hypothesized to occur if the lesion is either smaller than the resolution of the SPECT scan, located in regions near the calvarium or skull base that are obscured by the normally high thallium-201 uptake of the scalp, has a nonfocal location with leptomeningeal or subependymal spread [9], or is associated with tumor necrosis.

False-positives were present in our study and have been reported before in the literature.

There could be many reasons for this. First of all, our study did not look at individual lesions in assessing the accuracy of the scan but instead examined the diagnosis of individual patients. Thus, a patient may have had, for example, several lesions suspected to be considered positive for PCNSL, but only one that accumulated thallium-201. This scan would, however, still be considered positive for PCNSL, even though many of the lymphomatous lesions themselves were not “positive” for thallium-201. Also, scans were often performed after the initiation of anti-toxoplasmosis treatment, which may have reduced the parasite burden, making a true negative in toxoplasmosis patients more likely than if the scan were performed before the initiation of therapy.

A review of the literature reveals that studies with larger sample sizes seem to produce lower diagnostic efficiency results. Several studies with smaller sample sizes have shown remarkably high diagnostic accuracies [9-10]. However, another study that had a sample that was smaller but similar in size to the one utilized in our current study produced a similar relatively low diagnostic accuracy (71%) [11]. The reason for this is unknown.

Combining CSF EBV testing using PCR with thallium-201 SPECT has been reported to increase overall diagnostic accuracy and predictive value [18], and our study confirms this. Both toxoplasma serology [14] and gallium-67 SPECT [15-16], when individually coupled with thallium-201 SPECT imaging results, improve diagnostic accuracy. These tests may be utilized as adjuncts to thallium-201 SPECT scanning when results are equivocal or when a higher predictive value is desired by a clinician.

The limited sample size may be thought of as a limitation of this study; however, this is the largest study of its kind examining this highly selected patient population. In order to study a larger group of patients, a patient registry database may be advisable in the future.

### Quantification

In order for the thallium-201 SPECT brain imaging study to be clinically useful in the AIDS patient population, it should accelerate the diagnosis of PCNSL over the current method of waiting for a two to four week empiric trial of anti-toxoplasmosis therapy to be completed. Our study demonstrates that a positive thallium-201 SPECT scan has a relatively high positive predictive value with regards to the diagnosis of PCNSL, which suggests that patients with positive results could undergo empiric radiation treatment without resorting to brain biopsy. In such cases, few patients would benefit from an empiric trial of anti-toxoplasmosis therapy. Our data suggest a reconsideration of the current practice guidelines to include thallium-201 SPECT earlier in the diagnostic workup [17]. If the scan is positive, CSF EBV PCR may be used as corroboration with subsequent radiation therapy without brain biopsy [12]. Combining CSF EBV testing using PCR with thallium-201 SPECT scans increases overall diagnostic accuracy [18] and predictive value, providing clinicians with a more definitive answer to their diagnostic question. If the results of SPECT and EBV testing are discordant, then brain biopsy seems to be advisable.

However, if CSF cannot be safely obtained due to a risk of inevitable cerebral herniation, which is often the case with intracranial masses, we believe that these data still suggest that empiric radiation treatment should be considered in a patient with a positive thallium-201 SPECT. On the other hand, if a thallium-201 SPECT scan is negative, a trial of anti-toxoplasmosis therapy should be initiated. It must be kept in mind, however, that variation in the criteria used to read scans can alter results and that greater standardization needs to be established before we can reliably use thallium-201 SPECT imaging in widespread clinical practice. 

## Conclusions

The methodology we espouse in this paper for the overall approach to the AIDS patient with an intracranial mass lesion would avoid the morbidity and mortality of a brain biopsy. In addition, this would minimize any delay of treatment if the failure of a trial of anti-toxoplasmosis therapy is required before initiating radiation therapy.
